# Probiotics and Clostridium Difficile: A Review of Dysbiosis and the Rehabilitation of Gut Microbiota

**DOI:** 10.7759/cureus.5063

**Published:** 2019-07-02

**Authors:** Ashish S Kalakuntla, Gouthami Nalakonda, Kashyap Nalakonda, Chandra Varshini Pidikiti, Syed Ali Aasim

**Affiliations:** 1 Gastroenterology, Chalmeda Anand Rao Institute of Medical Sciences, Karimnagar, IND; 2 Gastroenterology, Kamineni Academy of Medical Sciences and Research Centre, Hyderabad, IND; 3 Anaesthesiology, Chalmeda Anand Rao Institute of Medical Sciences, Karimnagar, IND

**Keywords:** gut microbiota, antibiotic-associated diarrhea, lactobacillus, saccharomyces, probiotics, clostridium difficile-associated diarrhea

## Abstract

The basis of this paper is to address the use of probiotics as a novel approach to help treat the growing problem of antibiotic-associated diarrhea (AAD), particularly, Clostridium difficile-associated diarrhea (CDAD). Most of the available data regarding probiotics and their usefulness in treating Clostridium difficile infection (CDI) was collected and analyzed. Studies showed the effectiveness of probiotics in treating and also preventing CDI, as well as other gastrointestinal conditions such as Helicobacter pylori infection and inflammatory bowel disease. Probiotics also have, based on limited research, a comparatively minimal adverse effect profile and can aid in faster recovery from disease. Extensive research has been done on two organisms, Lactobacillus and Saccharomyces, but further research into other effective organisms are needed. More clinical trials also need to be conducted to better understand the side effect profile, optimal dosage, drug interactions, and long-term effects on gut microbiota.

## Introduction and background

A 72-year-old male was admitted to the hospital for severe right lobe consolidation of the lung and was receiving treatment with fluoroquinolones and intravenous therapy (IV). On his fourth day of admission, he complained of severe abdominal pain and was passing watery stools five times a day. His blood work showed increased leukocytes and his stool culture for the Clostridium difficile (C. difficile) toxin was positive. C. difficile, a type of antibiotic-associated diarrhea (AAD), should be considered a probable diagnosis for patients presenting with diarrhea while receiving treatment with antibiotics.

AAD is one of the frequent complications of antibiotic therapy worldwide. The most common antibiotic classes that cause AAD are fluoroquinolones and cephalosporins. It occurs due to the disruption of the habitual colonic gut flora due to prolonged use of antibiotics, which predisposes to diarrhea and infections by multiple pathogens [1]. Other risk factors can be dependent on associated comorbidities, history of previous hospitalizations, the severity of the infection, and advanced age [2-4]. C. difficile is an anaerobic, gram-positive, rod-shaped bacillus. It accounts for 15% - 25% of total AAD cases and is also the foremost cause of hospital-acquired bacterial infections, making it the most vicious of the enteric pathogens [5-8]. Clostridium difficile-associated diarrhea (CDAD) is a type of AAD which presents with a spectrum of disease entities ranging from daily watery stools to serious complications, such as toxic megacolon and, in severe cases, death. The main treatment for the infection is discontinuation of the causative antibiotics and initiating therapy through antibiotics, such as metronidazole or vancomycin. In recent years, however, there has been a significant increase in resistance due to the overuse of antibiotics and the emergence of new resistant strains of the bacteria [9]. Due to the increase in cases of CDAD resistant to antibiotic therapy, alternative approaches to antibiotics alone are being explored. One such approach is the use of probiotics to enhance the immunity of the host by replenishing gut microbiota and preventing colonization by resistant pathogens [10].

According to the Food and Agriculture Organization (FAO) of the United Nations and the World Health Organization (WHO), probiotics are “live microorganisms which, when administered in adequate amounts, confer a health benefit on the host” [11]. The use of probiotics as a viable treatment option has gained significant support recently with researchers believing it to be a more fitting alternative to cure gastrointestinal tract pathologies rather than trying to develop antibiotics that are more toxic and can cause a higher financial burden on society [12]. We are conducting this review, based on available studies, to analyze the efficacy of probiotics in treating cases of CDAD, discuss the side effect profile, and investigate the role that they may play in helping prevent recurrent cases of CDAD or other pathologies associated with dysbiosis of the gut microbiota. Two specific organisms, Lactobacillus and Saccharomyces, are emphasized. A focus is also laid on combination therapy with antibiotics, as opposed to antibiotic therapy alone, as a novel approach to treatment.

Method

A methodical search was conducted in the PubMed database and PubMed Central (PMC) using associated keywords from August 10 to August 22, 2018. The search results on specific keywords were as follows: 1) antibiotic-associated diarrhea, 4,139 results were displayed, 2) probiotics, 18,993 results were displayed, 3) Clostridium difficile and probiotics, 589 results were displayed, 4) synbiotics, 784 results were displayed, 5) Saccharomyces boulardii, 637 results were displayed, and 6) Lactobacillus, 34,347 results were displayed.

A total of 58,489 results were obtained using the keywords detailed above. The filters were then applied beginning with limiting the studies to the last five years, which showed 20,770 results. The studies were again limited to clinical trials and reviews, which showed 5,168 results. The results were further screened based on animal and human studies and 4,314 articles were obtained. After analyzing abstracts and full-text articles, we excluded duplicate and irrelevant data and selected 50 studies. After ruling out unrelated data, a total of 18 studies were included in this review.

Quality assessment (external validity) and ethical issues

All the information in the review has been obtained lawfully. Most of the data incorporated were written after the year 2000 and are peer-reviewed and acquired from journals on PubMed which have a journal impact factor > 1.

## Review

The therapeutic benefits of probiotic therapy in the case of CDAD have been explored in this discussion, along with a focus on two particular organisms, Lactobacillus and Saccharomyces.

In a systematic review, Valdés-Varela et al. claim that after a microbial imbalance in the gastrointestinal (GI) tract, probiotics can be an important tool in replenishing the intestinal microbiota [13]. They stated that particular strains, such as Lactobacillus rhamnosus GG (L. rhamnosus GG) and Saccharomyces boulardii (S. boulardii), have been studied extensively for their role in CDI and results show that their application in treating CDI is an encouraging prospect. In another study by Wilkins and Sequoia, it was shown that in cases of CDAD, ulcerative colitis, and acute infectious diarrhea, probiotics are novel therapeutic agents and that the efficacy of probiotics is dependent on the species, dose, and the duration of the therapy [14].

Valdovinos et al. studied C. difficile in the Mexican population and formulated 27 statements for the use of probiotics [15]. They formulated that probiotics cannot only be used for the treatment of AAD, like C. difficile, but also for other pathologies, such as Helicobacter pylori (H. pylori) infection and necrotizing enterocolitis. Probiotics can also be used to prevent recurrent flares of ulcerative colitis [15]. This study was specific only to the Mexican population; the data cannot be extrapolated to other populations and the inference is limited. Similarly, Cameron et al. revised randomized controlled trials in the region of Asia Pacific and found that S. boulardii and L. rhamnosus, when used along with oral rehydration therapy, showed improvement in pediatric gastroenteritis [16]. They also stated that these probiotics can be used for the prevention of CDI [16]. This study was again limited to a specific region and much inference cannot be sought. However, a point to be noted is that there was a curative effect of probiotics on two different populations with a different diet, genetic factors, and two different strains of infective pathogens.

A systematic review written by Shen et al. stated that probiotics showed an improved effect in the clinical setting when initiated within two days of starting antibiotics for treatment of C. difficile diarrhea [17]. They studied 19 articles that included 6,261 patients and the results showed the effectivity of probiotics with the p-value being 0.02. This study proves that probiotics can be used as an adjunct to antibiotic therapy, potentiating their action, and may lead to a faster recovery and decrease dependence on long-term antibiotic use. An article which studied many cohorts and randomized control trials stated that probiotics have been effective for the treatment of CDI, as well as other types of AAD and H. pylori infections [18]. One of the major risk factors for H. pylori infection is the long-term use of proton pump inhibitors (PPI), which are also regarded as a notable risk factor for CDI. Taking this into account, Lewis et al. conducted a retrospective cohort study that targeted proton pump inhibitor use and probiotics and concluded that decreasing PPI use and increasing probiotic use decreases the risk of hospital-acquired CDI [19].

In terms of their side effect profile, in a study by Wilkins and Sequoia, it was suggested that probiotic use is safe in all age groups, not including immunologically vulnerable patients [14]. Issa et al. agreed in a review and stated that according to most of the trials they studied, probiotics were benign and were associated with very minimal side effects [20]. A further clinical trial conducted by Goldenberg et al. showed that the use of probiotics, along with antibiotics, resulted in the reporting of less adverse effects compared to antibiotic therapy alone [21]. They stated that moderate evidence was present which showed that the number needed to benefit (NNTB) was 42 patients (95% CI: 32 to 58) where the NNTB was the number of patients that needed to be treated for one additional patient to benefit, to suggest that probiotics are a viable treatment option. In our opinion, this is a very good study as it was a meta-analysis of 31 randomized clinical trials which included 8,672 patients. However, there were a few reported cases of severe adverse effects, but the data was inconclusive and much inference cannot be sought.

In a systematic review, Szajewska et al. explored the preventive capacity of probiotics on AAD in children and strongly advocated for the use of S. boulardii for preventing AAD in children with risk factors, including previous episodes of AAD and hospitalization [22]. They also supported the use of S. boulardii for the prevention of CDAD. Pace et al. reached the same conclusion, finding that Lactobacilli, mainly L. rhamnosus GG, were not only very effective in treating recurrent C. difficile infections and other types of diarrhea but also that probiotics could be used as preventive medicine in healthy individuals [23].

Lactobacillus

A randomized controlled trial was conducted that included 33 participants, and they were given a daily dose of probiotics that contained L. acidophilus, L. paracasei, and Bifidobacterium. It was shown that diarrhea outcomes were improved in the participants who took probiotics when compared to the participants who were on a placebo [24]. L. reuteri with the pocR gene, when given with glycerol, become reuterin producers. Reuterin inhibits the growth of C. difficile and it is as potent as vancomycin. In a study conducted by Shinler et al., it was shown to be very effective when studied as antimicrobial agents [25]. Golić et al. conducted a study in which L. helveticus BGRA43, L. fermentum BGHI14, and Streptococcus thermophilus BGVLJ1-44 were used as probiotics against C. difficile and Clostridium perferingens [26]. It was shown that these probiotics induced inflammatory marker production against the pathogens, and they concluded that these have promising effects for the treatment of gut infections. In addition, an animal study was conducted by Ratsep et al. who concluded that using xylitol, along with Lactobacillus plantarum, decreased toxin formation by C. difficile and also stopped spores from germinating in the gut of hamsters that were on amoxicillin compared to the hamsters that did not receive synbiotics (p = 0.03) [27].

Saccharomyces

A prospective study was conducted on 163 patients in China, and it was found that S. boulardii decreased the incidence of AAD and also improved the symptoms (p < 0.05) [28]. The sample, however, was limited to only one country. In a separate randomized control study, Kabbani et al. noted that the subjects who were given the probiotic S. boulardii CNCM I-745 (SB), along with antibiotics, showed significant diminution in AAD due to less proliferation of pathogenic organisms, such as Escherichia coli (E. coli) [29]. The effectiveness of S. boulardii CNCM I-745 was confirmed by another report which cited various experimental and clinical studies and confirmed that this organism can be a useful tool in the management of AAD, H. pylori eradication, and also acute gastroenteritis [30]. 

## Conclusions

The application of probiotics in treating CDAD is an approach which has been known, but new interest has developed recently due to ongoing research in the field. After analyzing the available studies, most advocated in favor of using probiotics. However, comprehensive research is lacking, and studies need to be performed to better understand possible serious long-term complications and also to uncover other organisms which are potent in treating CDAD and other gastrointestinal pathologies.
